# Simultaneous metabolite MALDI-MSI, whole exome and transcriptome analysis from formalin-fixed paraffin-embedded tissue sections

**DOI:** 10.1038/s41374-022-00829-0

**Published:** 2022-08-31

**Authors:** Lisa Kreutzer, Peter Weber, Theresa Heider, Mathias Heikenwälder, Tobias Riedl, Philipp Baumeister, Frederick Klauschen, Claus Belka, Axel Walch, Horst Zitzelsberger, Julia Hess, Kristian Unger

**Affiliations:** 1 Research Unit Radiation Cytogenetics, Helmholtz Zentrum München, Neuherberg, Germany; 2 Clinical Cooperation Group Personalized Radiotherapy in Head and Neck Cancer, Helmholtz Zentrum München, Neuherberg, Germany; 3 Division of Chronic Inflammation and Cancer, German Cancer Research Center (DKFZ), Heidelberg, Germany; 4 Department of Otorhinolaryngology, University Hospital, LMU Munich, München, Germany; 5 Faculty of Medicine, Ludwig-Maximilians-University of Munich, Institute of Pathology, München, Germany; 6 Department of Radiation Oncology, University Hospital, Ludwig-Maximilians-University Munich, Munich, Germany; 7 German Cancer Consortium (DKTK), Partner Site Munich, and German Cancer Research Center (DKFZ), Heidelberg, Germany; 8 Research Unit Analytical Pathology, Helmholtz Zentrum München, Neuherberg, Germany

## Abstract

Matrix-assisted laser desorption ionization mass spectrometry imaging (MALDI-MSI) allows spatial analysis of proteins, metabolites, or small molecules from tissue sections. Here, we present the simultaneous generation and analysis of MALDI-MSI, whole-exome sequencing (WES), and RNA-sequencing data from the same formalin-fixed paraffin-embedded (FFPE) tissue sections. Genomic DNA and total RNA were extracted from (i) untreated, (ii) hematoxylin-eosin (HE) stained, and (iii) MALDI-MSI-analyzed FFPE tissue sections from three head and neck squamous cell carcinomas. MALDI-MSI data were generated by a time-of-flight analyzer prior to preprocessing and visualization. WES data were generated using a low-input protocol followed by detection of single-nucleotide variants (SNVs), tumor mutational burden, and mutational signatures. The transcriptome was determined using 3’-RNA sequencing and was examined for similarities and differences between processing stages. All data met the commonly accepted quality criteria. Besides SNVs commonly identified between differently processed tissues, FFPE-typical artifactual variants were detected. Tumor mutational burden was in the same range for tissues from the same patient and mutational signatures were highly overlapping. Transcriptome profiles showed high levels of correlation. Our data demonstrate that simultaneous molecular profiling of MALDI-MSI-processed FFPE tissue sections at the transcriptome and exome levels is feasible and reliable.

## Abstract (web summary)

The authors present a workflow that allows the simultaneous measurement of the whole exome and the transcriptome by next-generation sequencing from formalin-fixed paraffin-embedded tissue sections that were analyzed by matrix-assisted laser desorption ionization mass spectrometry imaging. The data and analyses demonstrate the feasibility and reproducibility of this approach, which expands the possibilities of multi-omics integration in cancer research.

## Introduction

In order to investigate the molecular makeup of tissues in an integrative fashion, multilevel “omics”-studies have gained great relevance^1–4^. Although desirable, it is challenging to assess multiple omics levels from the same tissue sample. However, with the advancement of omics methodology, it is now possible to generate high-quality genomic and transcriptomic profiles from small numbers of cells and nucleic acids of limited quality^5,6^. This paved the ground for performing different omics measurements from the same tissue specimen. Here, we present the data of matrix-assisted laser desorption ionization mass spectrometry imaging (MALDI-MSI), whole-exome sequencing (WES), and RNA sequencing (RNA-seq) generated from the same formalin-fixed paraffin-embedded (FFPE) tissue sections and demonstrate the feasibility and robustness of the applied protocols.

In order to investigate proteomics and metabolomics in an untargeted manner, mass spectrometry, which determines the mass-to-charge (*m/z*) ratio of ionized particles, is the method of choice^7,8^. MSI even extends the analytical capabilities of mass spectrometry by visualizing the spatial distribution of individual molecules within their histological context. MSI, which primarily is used to capture protein and metabolite levels, has been recently combined with approaches measuring additional molecular layers. Kazdal et al. described a multiplex approach that applied MSI with digital PCR to the same tissue sections^9^. The possibility of combining metabolic and genetic information from FFPE tissues was also investigated by combining MSI with fluorescence in situ hybridization^10^. MALDI-MSI can also be used to discriminate molecularly homogeneous regions within tissue sections prior to manual or laser microdissection for further molecular analyses^11^. To investigate clinical tissue samples at the genomic and transcriptomic levels, next-generation sequencing (NGS) has become state of the art and the simultaneous applicability of MALDI-MSI and NGS methodology on FFPE tissue sections would be highly desirable^4,12^.

Combining omics approaches has been done in numerous studies that integrated genomics, transcriptomics, proteomics, and metabolomics data generated from clinical samples^2,13^. However, so far, the feasibility of the generation of WES, RNA-seq, and MALDI-MSI data from the same FFPE tissue section has not been demonstrated.

In this technical note, we present a sequential coupling of MALDI-MSI with WES and RNA-seq on the same FFPE tissue section for three different specimens of head and neck squamous cell carcinoma (HNSCC).

## Material and methods

### Tumor tissue specimens

Tissue specimens of histologically confirmed HNSCC from three patients who had undergone surgical resection were provided in the framework of the Clinical Cooperation Group “Personalized Radiotherapy in Head and Neck Cancer” (Ludwig-Maximilians-University of Munich and Helmholtz Zentrum München). According to common pathology practice, the tissues were fixed with formalin for 24 h prior paraffin embedding. FFPE tissue sections (3 µm) were prepared using a microtome (HM340E, Thermo Scientific, Waltham, Massachusetts, USA) and mounted on indium tin oxide-treated poly-L-lysine-coated glass slides.

### MALDI-MSI

**Fig. 1:**
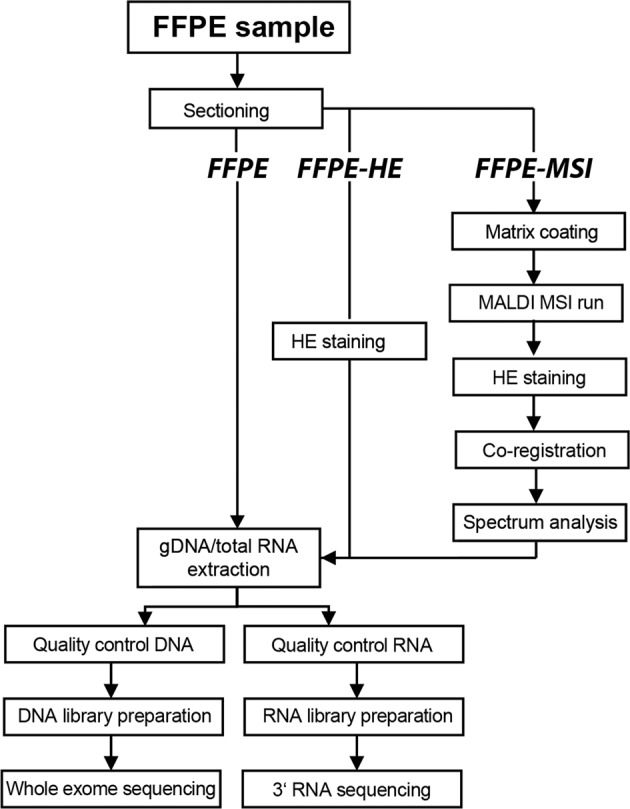
Workflow for RNA and exome sequencing on MALDI-MSI-processed FFPE tissue sections. For examinations of the effect of the MSI workflow on the genome and transcriptome, the FFPE samples were sectioned and subsequently processed in three different approaches. As a control approach, one sample was extracted directly after dissection (*FFPE*) and one sample was dissected and stained with hematoxylin and eosin (*FFPE-HE*). In the discovery approach, MSI with an additional HE staining was performed to follow the regular procedure of an MSI experiment (*FFPE-MSI*). After scanning the stained tissue for the MSI procedure, the same tissue was used for the extraction of gDNA and total RNA. Then the integrity of the nucleic acids was examined, the libraries were prepared and the samples were sequenced accordingly.

Three experimental setups for testing the effect of deparaffinization, hematoxylin and eosin staining, and MSI on RNA-seq and WES data were used (see Fig. 1). In the first, referred to as *FFPE*, the FFPE sections were deparaffinized by incubation for 60 min at 60 °C prior paraffin removal by two incubation steps in xylene for 7 min each. In the second, referred to as *FFPE-HE*, sections were deparaffinized prior to hematoxylin and eosin staining using a standard protocol^14^. In the third, referred to as *FFPE-MSI*, deparaffinized and HE-stained tissue sections were subjected to the MSI workflow. For this purpose, the deparaffinized section first was coated with a 9-aminoacridine (9-AA) ionization matrix. The matrix was prepared by dissolving 10 mg/ml 9-AA (Sigma-Aldrich, St. Louis, Missouri, USA) in 70% methanol. The tissue coating was performed using an automated spray system (Micro Fraction Collector, MALDI-Spotter SunCollect, SunChrom, Friedrichsdorf, Germany). Subsequently, mass spectrometric data were measured with an Ultraflex III MALDI-TOF instrument (Bruker Daltonics, Bremen, Germany) in linear negative mode, detecting metabolites mainly in the mass range of 100–1060 Da and a lateral resolution of 60 µm. The tissue sections were stained with hematoxylin and eosin, scanned with a digital slide scanning system (Mirax Desk, Carl Zeiss MicroImaging, Göttingen, Germany), and co-registered with the MALDI-MSI measurement in the FlexImaging software (Bruker Daltonics, Bremen, Germany), to match the MS data with the histological features of the tissue sections. Metabolites likely to be reflected by the *m/z* species were identified by requesting the Human Metabolome Database (HMDB) with an allowed tolerance of 10 ppm.

### Next-generation sequencing

Genomic DNA and total RNA were isolated and purified simultaneously from the whole tissue sections using the AllPrep DNA/RNA FFPE Kit according to the manufacturer’s protocol (Qiagen, Hilden, Germany), followed by assessing nucleic acid integrity with the Agilent 2100 Bioanalyzer (Agilent Technologies, Santa Clara, California, USA) and the Agilent DNA 12000 Assay Kit or the Agilent RNA 6000 Pico Assay Kit, respectively.

#### Whole-exome sequencing

In order to determine the fragmentation status and further amplification potential of the extracted gDNA, the TruSeq FFPE DNA Library Prep QC Kit (Illumina, San Diego, California, USA) was used according to the manufacturer’s protocol and executed on a ViiA 7 Real-Time PCR System (Thermo Scientific, Waltham, Massachusetts, USA). This quality assessment approach is based on a genomic qRT-PCR, where the Ct values (cycle thresholds) of the tissue sample DNAs are set in relation to the Ct of a validated amplicon control DNA (proprietary). Depending on the obtained ratio, the samples were classified into different quality levels which determine the input quantity for the DNA library preparation. The SureSelect XT v6 (target size: 60 Mb) Target Enrichment System (Agilent Technologies, Santa Clara, California, USA) was used to construct a whole exome library which was subsequently sequenced on a HighSeq4000 system (Illumina, San Diego, California, USA) according to the manufacturer’s protocol.

#### 3’ mRNA sequencing

Total RNA integrity was assessed by means of the DV200 value (i.e., the percentage of fragments >200 nucleotides) using an Agilent RNA 6000 Pico Chip Kit on the Agilent 2100 Bioanalyzer (Agilent Technologies, Santa Clara, California, USA). 3’ mRNA sequencing libraries were generated using the QuantSeq 3’ mRNA-Seq Library Prep Kit (Lexogen, Vienna, Austria) according to the manufacturer’s instructions. For library amplification, PCR cycles were determined using the PCR Add-on Kit for Illumina (Lexogen, Vienna, Austria). Before sequencing on a HighSeq4000 system (Illumina, San Diego, California, USA), quantity and quality of sequencing libraries were assessed using the Quanti-iT PicoGreen dsDNA Assay Kit (Thermo Scientific, Waltham, Massachusetts, USA) and the Bioanalyzer High Sensitivity DNA Analysis Kit (Agilent Technologies, Santa Clara, California, USA).

### Bioinformatic analyses

#### Single-nucleotide variants and genomic copy number changes

The WES profiles were preprocessed and analyzed according to the GATK best practices workflow “Somatic short variant discovery” (https://gatk.broadinstitute.org/hc/en-us/articles/360035894731-Somatic-short-variant-discovery-SNVs-Indels-). Implementation of the code was mainly adapted from the GATK workflows GitHub repository (https://github.com/gatk-workflows). Before applying the gatk4-data-processing workflow, the raw fastq files from different sequencing lanes were concatenated using the “cat” bash command. The unaligned bam files were then subjected to alignment using bwa-mem, Mutect2 calling of SNVs, and indels. GATK4-Mutect2 was run in tumor-only mode. SNVs which were quality-flagged by Mutect2 were filtered out. The resulting vcf files were imported into R and further processed using functions of the maftools R-Bioconductor package^15^. In addition, only SNVs were kept that were covered by more than 10 reads. Furthermore, the variants had to be present as coding or noncoding variant in the Cosmic (version 92) database and, furthermore, their population allele frequency (any ethnicity) was not allowed to exceed 10^–5^ according to the gnomAD database (https://gnomad.broadinstitute.org). Furthermore, CNVs of the top 100 frequently mutated genes were excluded^16^. The tumor mutational burden (TMB) was calculated as the ratio between the number of called variants per megabase. For calling mutational signatures with functions of the deconstructSigs R package, the Cosmic SBS signatures v3 (https://cancer.sanger.ac.uk/cosmic/signatures) were used.

#### Analysis of RNA-sequencing data

The RNA-seq data were preprocessed as follows: the forward-reads of the 150 bp paired-end sequencing were subjected to adapter trimming using BBDuk (https://jgi.doe.gov/data-and-tools/bbtools/bb-tools-user-guide/bbduk-guide/), followed by alignment to human genome 38 (hg38) using the STAR aligner. Aligned reads were counted by htseq-count (https://htseq.readthedocs.io/en/release_0.11.1/count.html). The count files were then imported into R for further processing using the R DESeq2 package^17^. The counts per Ensembl transcript id were summarized per gene and the names were converted to HGNC gene symbols. Only genes with a total average of 10 counts per profile were kept in the dataset. For pairwise-correlation (Spearman) and visualization by scatter plots the profiles were vst-transformed.

#### Quality assessment of sequencing data

For both, WES and RNA-seq, quality assessment of raw and mapped reads was performed using FastQC and the results were summarized and visualized using MultiQC.

### Matrix-associated add-on experiment

In order to assess the potential impact of the matrix on the results of the sequencing approaches, additional matrix-based analyses were performed. For this purpose, an approach without prior matrix application, an approach with prior matrix application, and an approach in which the process of washing off the matrix after the MSI run was simulated were carried out (see Supplementary Fig. S1). A 9-AA matrix was used, which was sprayed on as described above. Furthermore, in order to estimate the minimum amount of sample material required for successful amplification of the RNA and subsequent sequencing, a sample consisting of 1/2, 1/4, or 1/8 tissue was included from the approach with removed matrix in addition to the complete tissue isolate. Extraction of total RNA was performed with the RNeasy FFPE Kit (Qiagen GmbH, Hilden, Deutschland) with slight modifications. To increase the digestion activity of proteinase K on contaminating proteins and endogenous nucleases, samples were incubated overnight at 56 °C. To remove all residues of salts, the RPE buffer washing volume was increased to 500 µl in two successive washing steps. For analysis of nucleic acid integrity, reverse transcription was performed on the RNA. The SuperScript VILO cDNA synthesis Kit (Thermo Scientific, Waltham, Massachusetts, USA) was applied according to the manufacturer’s protocol and subjected to qRT-PCR (ViiA 7 Real-Time PCR System, Life Technologies, Carlsbad, California, USA) using TaqMan gene expression assays (Thermo Scientific, Waltham, Massachusetts, USA) against the housekeeping genes actin beta (ACTB, assay ID: Hs01060665_g1, amplicon length: 63 bp), glyceraldehyde-3-phosphate dehydrogenase (assay ID: Hs99999905_m1, amplicon length: 122 bp) and phosphoglycerate kinase 1 (PGK1, assay ID: Hs99999906_m1, amplicon length: 75 bp). Assessment of RNA integrity and 3’ mRNA sequencing was performed as described above.

## Results and discussion

The coupling of omics methods that capture different molecular layers from the same biological sample is the prerequisite of integrative multilevel integration approaches. Here, we aimed to test the feasibility of determining the spatially resolved metabolite level using MALDI-MSI, the genome level using WES, and the transcriptome level using RNA-seq from the same FFPE tissue sections. For each molecular level, the quantity, quality, and plausibility of the resulting data were evaluated and compared for three HNSCC FFPE tissue samples, each of which was *FFPE*, *FFPE-HE*, and *FFPE-MSI* processed (Fig. 1).

### MALDI-MSI at the metabolite level

**Fig. 2:**
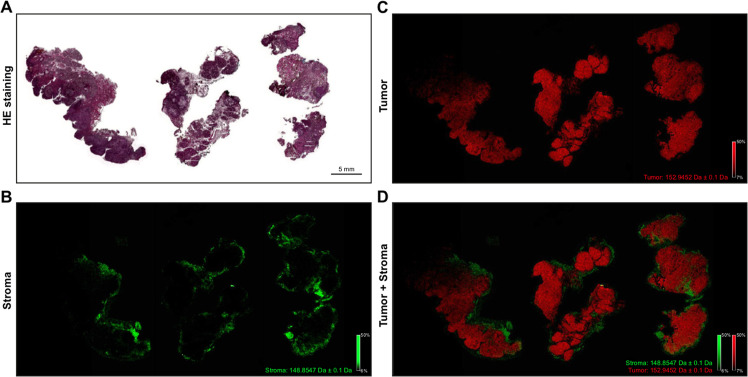
Spatial distribution of specific *m/z* species. By matching the FFPE-HE staining **A** with the MSI measurement, specific tumor tissue areas can be visualized. The metabolite *m/z* 148.8547 is exclusively abundant in the tumor stroma **B**, whereas *m/z* 152.9452 is clearly overrepresented in tumor cell areas **C**. The overlay of the two visualizations **D** shows that the metabolites do not overlap in any area.

The MSI measurement at a local resolution of 60 µm resulted in a total number of 89,685 spectra. All spectra were detected in a mass range between *m/z* 100 and 1060 to include the common metabolic masses^18^. The calibration was performed using phosphorus red. The overall spectra were comparable with regard to mass signals. Sample 1 contained 347, sample 2 contained 344, and sample 3 contained 377 mass signals after peak picking, based on a signal-to-noise ratio of 2. As expected, the peak of mass *m/z* 193.092, which corresponds to the molecular weight of the matrix substance 9-AA, formed the maximum of all components present in the spectrum with an intensity of 83.69. An HMDB database request with the identified *m/z* species revealed 772 metabolites for tissue sample 1, 1314 metabolites for sample 2, and 990 metabolites for sample 3. The three tissue samples shared 296 database-revealed metabolites (SI File 1). To ensure plausibility of the MSI measurement, metabolites with specific abundances in the tumor (*m/z* 152.9452) and stroma (*m/z* 148.8547), respectively, were visualized (Fig. 2). The histological composition of the tumor and tumor stroma was well reflected by these two *m/z* species. This, together with the quantitative QA measures demonstrates the plausibility of the MALDI-MSI data generated.

### Quality of genomic DNA

Following the qRT-PCR run, baseline correction was performed on the generated data and the average Ct value of the amplicon control DNA (ACD1) was subtracted from the Ct value of the samples. This corrected ΔCt value was used to classify the samples into the following different quality levels: ΔCt value between –1 and 1: “high quality”, recommended input quantity: 10 ng; ΔCt value between 1.0 and 2.5: “medium quality”, recommended input quantity: 20–50 ng; ΔCt value between 2.5 and 4.0: “low quality”, recommended input quantity: 50–100 ng. The input quantity for DNA library preparation was adjusted according to this classification. Tissue 1 had mean ΔCTs of 1.26 (*FFPE*), 0.88 (*FFPE-HE*), and 0.96 (*FFPE-MSI*), respectively. The untreated sample of tissue 1 was classified as medium quality, and the HE-stained and MSI-processed samples as good quality. Tissue 2 achieved averaged ΔCT values of 7.40 (*FFPE*), 6.74 (*FFPE-HE*), and 6.71 (*FFPE-MSI*) classifying all as worse than “low quality”. Tissue 3 showed values of 2.97 (FFPE), 3.31 (FFPE-HE), and 3.00 (FFPE-MSI) that classifies them as “low quality”. Although some samples were out of the manufacturer’s recommended range, we decided not to exclude any because all samples showed detectable amplification. Hence, we subjected all available DNA (tissue 1: 508.0 ng *FFPE*, 504.0 ng *FFPE-HE*, and 500.0 ng *FFPE-MSI*; tissue 2: 152.8 ng *FFPE*, 100.0 ng *FFPE-HE* and 191.2 ng *FFPE-MSI*; and tissue 3: 114.4 ng *FFPE*, 149.2 ng *FFPE-HE* and 172.8 ng *FFPE-MSI*) to exome library preparation.

### Quality of total RNA

The quality of the RNA was assessed by evaluating the DV200 values. According to the percentage of fragments with a size greater than 200 nucleotides, the quality of RNA integrity is classified as high (>70%), medium (50–70%), or low (30–50%) quality or rather too degraded (<30%), respectively. Accordingly, the *FFPE* (15%), *FFPE-HE* (18%), and *FFPE-MSI* (19%) samples of tissue 1 were classified as too degraded. Tissue 2 with DV200 values just below 50 (*FFPE*: 48%, *FFPE-HE*: 49%, *FFPE-MSI*: 48%) is in the low-quality range and tissue 3 with values (*FFPE*: 31%, *FFPE-HE*: 27%, *FFPE-MSI*: 26%) around 30 is between low RNA quality and too degraded samples. The RNA integrity of the samples is in a lower range, which was to be expected based on the empirical values for RNA with FFPE processing. However, since all samples had fragments in the range >200 nucleotides and the manufacturer’s quality assessment only addressed hypothetical chances of success, all samples were subjected to RNA-seq library preparation.

### Whole-exome sequencing raw and processed data quality

WES of the nine samples yielded 77.3 million reads on average and ranged between 62.8 million (tissue 1, *FFPE*) and 94.8 million (tissue 3, *FFPE-MSI*) paired-end reads. All sequences had a length of 101 bp. Average duplicate rate was 49.27%, ranging between 31.9% (tissue 3, *FFPE*) and 80.2% (tissue 2, *FFPE-MSI*). The GC-content of samples was 52.9% in average and ranged between 51.0% (tissue 2, *FFPE*) and 55.0% (tissue 3, *FFPE-HE*/*FFPE-MSI*). Quality scores (phred) of all sequences were greater than 30. The percentage of overrepresented sequences was below 2% in all samples. The mean mapping rate after read alignment using Bowtie 2 against the human reference genome was 96.30%, ranging from 73.21% (tissue 1, *FFPE-HE*) to 99.89% (tissue 3, *FFPE-MSI*).

**Fig. 3:**
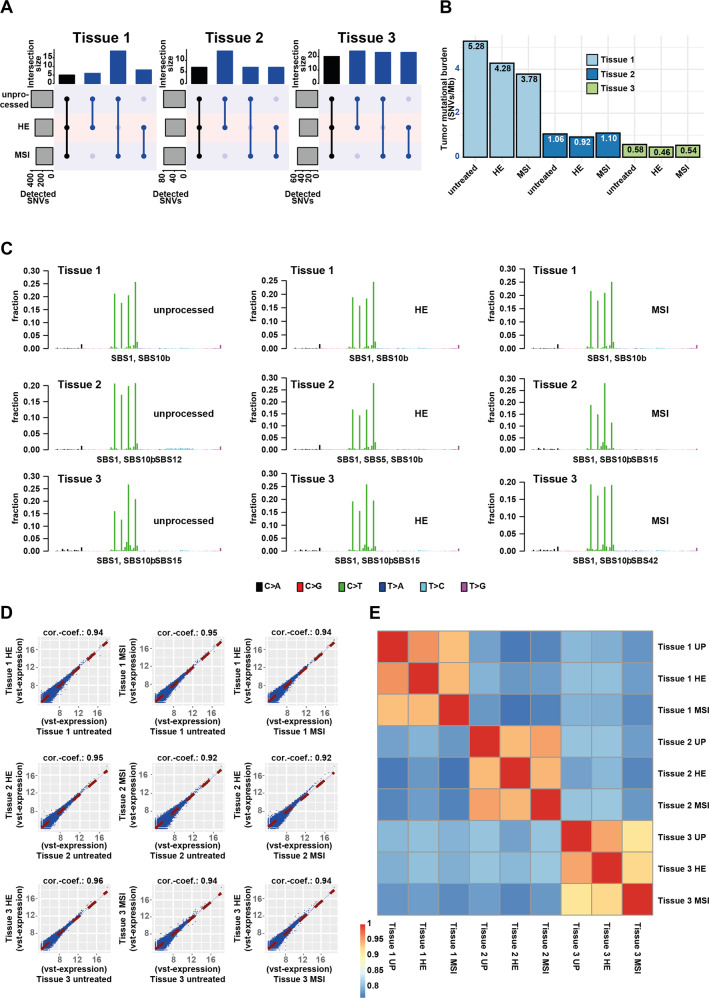
Comparative analysis of WES and transcriptome profiles of FFPE, FFPE-HE, and FFPE-MSI analyzed tissues. **A** Numbers of overlapping SNVs detected after differential processing in the three tissues. **B** Bar plots of the tumor mutational burdens detected in all samples. **C** Top 30 frequent SNVs and affected genes per tissue and sample. **D**, **E** Pairwise correlations between gene expression profiles of all combinations of processing steps for the three tissues analyzed visualized as scatter plots (**D**) and a correlation heatmap (**E**).

We determined the number of SNVs per tissue that were common and unique between the different treatments. After filtering, in total 1474 variants were detected—of these 1081 SNVs were detected in tissue 1 (*FFPE*: 416, *FFPE-HE*: 341, and *FFPE-MSI*: 324), 232 in tissue 2 (*FFPE*: 80, *FFPE-HE*: 73 and *FFPE-MSI*: 79) and 161 in tissue 3 (*FFPE*: 58, *FFPE-HE*: 49, and *FFPE-MSI*: 54). The extent of SNVs common between treatments differed strongly between the three tissues. In tissue 1, 5 SNVs were common between all treatments, 6 between *FFPE* and *FFPE-HE*, 8 between *FFPE-HE* and *FFPE-MSI,* and 19 between *FFPE* and *FFPE-MSI*. In tissue 2, 7 SNVs were common between all treatments, 14 between *FFPE* and *FFPE-HE*, 7 between *FFPE-HE* and *FFPE-MSI,* and 7 between *FFPE* and *FFPE-MSI*. In tissue 3, 20 SNVs were common between all treatments, 24 between *FFPE* and *FFPE-HE*, 23 between *FFPE-HE* and *FFPE-MSI,* and 23 between *FFPE* and *FFPE-MSI*. From the commonly detected SNVs in tissue 1, 3 out of 23 (13%) are known Cancer Gene Census (CGC) genes and 20 genes (87%) appeared in PubMed entries associated with the keyword “cancer”. For tissue 2 with 14 common genes, 3 (21%) were known CGC genes and 11 (79%) were published in the context of cancer. In tissue 3, 30 common genes were detected, while 2 (7%) were CGC-reported and 26 (87%) appeared in cancer-associated publications. A detailed representation of the data can be found in Supplementary Table S2 and Fig. 3A.

The TMB, reflecting the percentage of the genome covered by the whole exome analysis that is affected by SNVs, was 4.4% on average for the treatments of tissue 1 while the *FFPE* sample had a TMB of 5.28%, *FFPE-HE* 2.28%, and *FFPE-MSI* 3.78%. Tissue 2 had a mean TMB of 1.3%, with the *FFPE* sample showing 1.06%, *FFPE-HE* 0.92%, and *FFPE-MSI* 1.10%. Tissue 3 had a mean TMB of 0.53%, with the *FFPE* sample showing 0.58%, *FFPE-HE* 0.46%, and *FFPE-MSI* 0.54% (Fig. 3B). The TMB detected in our samples is well in the range as recently reported median TMB of 2.079 in HNSCC^19^.

Furthermore, we compared the mutational signatures (single base substitutions [SBS]) between the differently treated tissues. Tissue 1 only showed SBS1 and SBS10b in all treatments. Tissue 2 showed SBS1 and SBS10b in all treatments plus SBS12 in the *FFPE*, SBS5 in the *FFPE-HE,* and SBS15 in the *FFPE-MSI* sample. For tissue 3, SBS1 and SBS10b were detected and in addition to this, SBS15 in the *FFPE* and the *FFPE-HE* sample and SBS42 in the *FFPE-MSI* sample. Except SBS12, all signatures are reported as being frequently affected in HNSCC in the descriptions of the signatures given on the Sanger Cosmic website https://cancer.sanger.ac.uk/signatures/sbs.

### RNA-sequencing raw and processed data quality

Quantseq 3’-RNA sequencing of the nine samples averaged 6.73 million (M) uniquely mapped reads (range: 4.5–9.5 M uniquely assigned reads). In detail, tissue 1/*FFPE* showed 9.5 M (84% alignment), tissue 1/*FFPE-HE* 8.1 M (82%), tissue 1/*FFPE-MSI* 6.3 M (83%), tissue 2/*FFPE* 6.6 M (81%), tissue 2/*FFPE-HE* 6.6 M (77%), tissue 2/*FFPE-MSI* 8.2 M (80%), tissue 3/*FFPE* 4.5 M (75%), tissue 3/*FFPE-HE* 5.4 M (84%), and tissue 3/*FFPE-MSI* 5.4 M (83%) aligned reads. When comparing the similarity of the expression profiles using Spearman correlation all pairwise comparisons between the differently treated samples of the samples showed correlation coefficients greater than 91% (Fig. 3D)

In hierarchical clustering analysis of the Euclidean distance of the log-transformed (variance stabilized transformation) expression values, all samples belonging to the same tissue clustered together (Fig. 3E).

Hierarchical clustering of the distance matrix (Euclidean) from log-transformed expression values, which were built from read counts per gene by variance transformed transformation, (vst) showed co-clustering of expression profiles from the same tissues. The expression of the X-inactive specific transcript gene was consistent with the sex of the patients, which, together with the overall high correlation between profiles from the same tissues and the low correlation between patients, demonstrates the good feasibility and plausibility of using 3’-RNA-seq.

In conclusion, we show that method coupling of MALDI-MSI, WES, and RNA-seq is feasible and can generate technically sound and plausible data. As typical for whole exome data generated from FFPE tissues in tumor-only mode, a high proportion of technically artificial variants were detected. This limitation could be addressed by including matched normal tissue samples for more accurate detection of SNVs.

## Supplementary information

Supplementary Figures and Tables

## Data Availability

The datasets used and/or analyzed during the current study are available from the corresponding author on reasonable request.
