# Supplementary material for: Simultaneous metabolite MALDI-MSI, whole exome and transcriptome analysis from formalin-fixed paraffin-embedded tissue sections
Source: Lab Invest. 2022 Aug 31;102(12):1400–5. doi: 10.1038/s41374-022-00829-0 (PMC9708593; doi:10.1038/s41374-022-00829-0)
Supplement: Supplementary file 1 — Supplementary Figures and Tables [file 41374_2022_829_MOESM1_ESM.docx]

**Simultaneous metabolite MALDI-MSI, whole exome and transcriptome analysis from formalin-fixed paraffin-embedded tissue sections**

Supplemental information

**Figure S1: Experimental treatment scheme of tissue samples**. One tissue section was left unprocessed as positive control (*without matrix*), while two further sections were coated with matrix. From one of the coated sections the matrix was washed off according to the usual MSI workflow (*washed off matrix*), while from the other section the RNA was extracted with remaining matrix coating (*with matrix*). One part of the extracted RNA was translated into cDNA for quality analysis and then examined for integrity using qRT-PCR. The other part of the RNA was used for library preparation and subsequent 3' sequencing.

**Figure S2: SBS mutational signature profiles of each sample after WES.** The mutational patterns for each sample are shown while every position on the x-axis gives the fraction (height of bars) of a particular mutational pattern within all mutational patterns. The bars are colored according to the type of base substitution. The subtitle contains the most abundant detected SBS signatures per tissue.

**Figure S1**

**Figure S2**

**Table S1:** Quality assessment of isolated DNA and RNA

| **Sample** | **DNA** | | **RNA** | |
| --- | --- | --- | --- | --- |
|  | **c [ng/µl]** | **Ct value** | **c [ng/µl]** | **DV200 [%]** |
| Tissue 1 FFPE | 25.40 | 22.76 | 117.00 | 15 |
| Tissue 1 FFPE-HE | 25.20 | 22.38 | 71.60 | 18 |
| Tissue 1 FFPE-MSI | 25.00 | 22.45 | 99.40 | 19 |
| Tissue 2 FFPE | 7.64 | 28.90 | 62.80 | 48 |
| Tissue 2 FFPE-HE | 5.00 | 28.24 | 57.00 | 49 |
| Tissue 2 FFPE-MSI | 9.56 | 28.21 | 65.00 | 48 |
| Tissue 3 FFPE | 5.72 | 24.46 | 36.20 | 31 |
| Tissue 3 FFPE-HE | 7.46 | 24.81 | 34.00 | 27 |
| Tissue 3 FFPE-MSI | 8.64 | 24.50 | 22.20 | 26 |

c: concentration assessed by Qubit fluorometer (Thermo Scientific, Waltham, Massachusetts, USA)

ct: cycle threshold detected for accelerated cell death 1 (ACD1) gene by qRT-PCR using the TruSeq FFPE DNA Library Prep QC Kit (Illumina, San Diego, California, USA)

**Table** **S2:** SNV consensus Tissue 1

| **HUGO symbol** | **Chromo-some** | **Start position** | **End position** | **Reference allele** | **Tumor seq allele** | **Variant classification** | **Cancer gene census** | **Untreated vs. FFPE-HE-MSI** | **Untreated vs. FFPE-HE** | **FFPE-HE vs. FFPE-HE-MSI** | **PubMed counts:**  **HUGO symbol AND cancer** |
| --- | --- | --- | --- | --- | --- | --- | --- | --- | --- | --- | --- |
| PPP2R2B | chr5 | 146701056 | 146701056 | G | A | Nonsense mutation | no | x | x | x | 26 |
| GOLGA8R | chr15 | 30407956 | 30407956 | A | G | Missense mutation | no | x | x | x | 0 |
| TP53 | chr17 | 7675084 | 7675084 | G | T | Nonsense mutation | yes | x | x | x | 19203 |
| THAP8 | chr19 | 36039519 | 36039519 | C | T | Missense mutation | no | x | x | x | 0 |
| SCUBE2 | chr11 | 9033755 | 9033755 | G | A | Missense mutation | no | x | x | x | 33 |
| HSPA6 | chr1 | 161524937 | 161524937 | C | T | Silent mutation | no | x |  |  | 47 |
| NT5DC2 | chr3 | 52533601 | 52533601 | A | G | Missense mutation | no | x |  |  | 5 |
| PIK3CA | chr3 | 179218286 | 179218286 | C | G | Missense mutation | yes | x |  |  | 5107 |
| HTR1A | chr5 | 63960553 | 63960553 | G | A | Silent mutation | no | x |  |  | 19 |
| CORO7 | chr16 | 4364779 | 4364779 | C | T | Missense mutation | no | x |  |  | 1 |
| HIPK4 | chr19 | 40380963 | 40380963 | C | T | Missense mutation | no | x |  |  | 3 |
| KIR2DL1 | chr19 | 54783517 | 54783517 | A | G | Silent mutation | no | x |  |  | 116 |
| ARVCF | chr22 | 19981966 | 19981966 | G | A | Silent mutation | no | x |  |  | 31 |
| GRIPAP1 | chrX | 48997338 | 48997338 | C | T | Missense mutation | no | x |  |  | 6 |
| SCN1A | chr2 | 166015680 | 166015680 | G | A | Silent mutation | no | x |  |  | 25 |
| FOXP1 | chr3 | 71130603 | 71130603 | C | G | Intron mutation | yes | x |  |  | 349 |
| HSPA1A | chr6 | 31815720 | 31815720 | A | G | 5'UTR mutation | no | x |  |  | 132 |
| TMEM191C | chr22 | 21468303 | 21468303 | C | T | Intron mutation | no | x |  |  | 0 |
| LRRC8E | chr19 | 7898627 | 7898627 | A | G | Intron mutation | no | x |  |  | 3 |
| GOSR1 | chr17 | 30563343 | 30563343 | G | A | Intergenic region mutation | no |  | x |  | 8 |
| PFKL | chr21 | 44316247 | 44316247 | C | T | Missense mutation | no |  |  | x | 20 |
| PCMTD1 | chr8 | 51817881 | 51817881 | T | C | 3'UTR mutation | no |  |  | x | 6 |
| LRTOMT | chr11 | 72109304 | 72109304 | C | T | 3'UTR mutation | no |  |  | x | 1 |

**Table** **S3:** SNV consensus Tissue 2

| **HUGO symbol** | **Chromo-some** | **Start position** | **End position** | **Reference allele** | **Tumor seq allele 2** | **Variant classification** | **Cancer gene census** | **Untreated vs. FFPE-HE-MSI** | **Untreated vs. FFPE-HE** | **FFPE-HE vs. FFPE-HE-MSI** | **PubMed counts:**  **HUGO symbol AND cancer** |
| --- | --- | --- | --- | --- | --- | --- | --- | --- | --- | --- | --- |
| GOLGA8R | chr15 | 30407956 | 30407956 | A | G | Missense mutation | no | x | x | x | 0 |
| VPS52 | chr6 | 33267271 | 33267271 | C | T | Missense mutation | no | x | x | x | 10 |
| NUMA1 | chr11 | 72006231 | 72006231 | G | A | Silent mutation | yes | x | x | x | 107 |
| CLEC1B | chr12 | 9998321 | 9998321 | C | T | Missense mutation | no | x | x | x | 12 |
| VIPAS39 | chr14 | 77435872 | 77435872 | G | A | Missense mutation | no | x | x | x | 3 |
| MZT2A | chr2 | 131491116 | 131491116 | G | A | Intron mutation | no | x | x | x | 0 |
| LILRB3 | chr19 | 54222998 | 54222998 | C | G | 5'UTR mutation | no | x | x | x | 8 |
| FAM136A | chr2 | 70297444 | 70297444 | C | G | Missense mutation | no |  | x |  | 4 |
| ZNF638 | chr2 | 71423020 | 71423020 | C | G | Missense mutation | no |  | x |  | 4 |
| RBM47 | chr4 | 40438573 | 40438573 | G | A | Silent mutation | no |  | x |  | 17 |
| GALNT17 | chr7 | 71415981 | 71415981 | C | T | Missense mutation | no |  | x |  | 0 |
| TP53 | chr17 | 7674262 | 7674262 | T | C | Missense mutation | yes |  | x |  | 19203 |
| ZNF285 | chr19 | 44386880 | 44386880 | T | C | Silent mutation | no |  | x |  | 0 |
| TP53 | chr17 | 7673700 | 7673700 | C | G | Splice site mutation | yes |  | x |  | 19203 |

**Table S4**: SNV consensus Tissue 3

| **HUGO symbol** | **Chromo-some** | **Start position** | **End position** | **Reference allele** | **Tumor seq allele 2** | **Variant classification** | **Cancer gene census** | **Untreated vs. FFPE-HE-MSI** | **Untreated vs. FFPE-HE** | **FFPE-HE vs. FFPE-HE-MSI** | **PubMed counts:**  **HUGO symbol AND cancer** |
| --- | --- | --- | --- | --- | --- | --- | --- | --- | --- | --- | --- |
| NT5DC2 | chr3 | 52533601 | 52533601 | A | G | Missense mutation | no | x | x | x | 5 |
| IFI16 | chr1 | 159020442 | 159020442 | A | C | Missense mutation | no | x | x | x | 122 |
| RARRES1 | chr3 | 158732380 | 158732380 | C | G | Missense mutation | no | x | x | x | 57 |
| MUC4 | chr3 | 195754276 | 195754276 | C | T | Missense mutation | yes | x | x | x | 578 |
| NCAPG | chr4 | 17824997 | 17824997 | G | A | Silent mutation | no | x | x | x | 51 |
| MSH5 | chr6 | 31753355 | 31753355 | C | T | Silent mutation | no | x | x | x | 53 |
| RPP25L | chr9 | 34611067 | 34611067 | C | T | Missense mutation | no | x | x | x | 0 |
| PIGO | chr9 | 35091619 | 35091619 | C | T | Silent mutation | no | x | x | x | 5 |
| ZBTB5 | chr9 | 37441782 | 37441782 | G | A | Missense mutation | no | x | x | x | 4 |
| NUP188 | chr9 | 129002939 | 129002939 | G | A | Silent mutation | no | x | x | x | 8 |
| KCNT1 | chr9 | 135765144 | 135765144 | C | T | Silent mutation | no | x | x | x | 4 |
| COA4 | chr11 | 73873324 | 73873324 | C | T | Missense mutation | no | x | x | x | 0 |
| OR10G9 | chr11 | 124023748 | 124023748 | G | C | Missense mutation | no | x | x | x | 1 |
| CST8 | chr20 | 23492979 | 23492979 | C | T | Missense mutation | no | x | x | x | 4 |
| PRDM15 | chr21 | 41839798 | 41839798 | G | A | Silent mutation | no | x | x | x | 7 |
| ZBTB20 | chr3 | 114350721 | 114350721 | C | T | Missense mutation | no | x | x | x | 36 |
| RARRES1 | chr3 | 158732509 | 158732509 | C | G | 5'Flank mutation | no | x | x | x | 57 |
| CNTNAP3B | chr9 | 41953398 | 41953398 | C | T | Intron mutation | no | x | x | x | 2 |
| TP53 | chr17 | 7674857 | 7674857 | A | G | Splice site mutation | yes | x | x | x | 19203 |
| GABRE | chrX | 151974644 | 151974644 | G | A | 5'UTR mutation | no | x | x | x | 19 |
| GOLGA8R | chr15 | 30407956 | 30407956 | A | G | Missense mutation | no | x |  |  | 0 |
| LSG1 | chr3 | 194652735 | 194652735 | G | A | Silent mutation | no | x |  |  | 1 |
| PIDD1 | chr11 | 802748 | 802748 | C | T | Missense mutation | no | x |  |  | 46 |
| RARRES1 | chr3 | 158732389 | 158732389 | A | G | Silent mutation | no |  | x |  | 57 |
| ATP2A3 | chr17 | 3941136 | 3941136 | C | T | Silent mutation | no |  | x |  | 28 |
| SLC25A41 | chr19 | 6432089 | 6432089 | G | A | Missense mutation | no |  | x |  | 1 |
| TMEM191C | chr22 | 21468303 | 21468303 | C | T | Intron mutation | no |  | x |  | 0 |
| CCDC183 | chr9 | 136799852 | 136799852 | T | G | Intron mutation | no |  |  | x | 2 |
| CFAP46 | chr10 | 132867532 | 132867532 | C | T | Intron mutation | no |  |  | x | 3 |
| MED24 | chr17 | 40020363 | 40020363 | G | A | Intron mutation | no |  |  | x | 10 |
